# Anthropometric Markers and Iron Status of 6–12-Year-Old Thai Children: Associations and Predictors

**DOI:** 10.1155/2021/9629718

**Published:** 2021-04-13

**Authors:** Orapa Suteerojntrakool, Tharida Khongcharoensombat, Sirinuch Chomtho, Chansuda Bongsebandhu-phubhakdi, Therdpong Tempark, Mary Fewtrell

**Affiliations:** ^1^Ambulatory Division, Department of Paediatrics, Faculty of Medicine, Chulalongkorn University, Bangkok 10330, Thailand; ^2^Paediatric Nutrition Research Unit, Division of Nutrition, Department of Paediatrics, Faculty of Medicine, Chulalongkorn University, Bangkok 10330, Thailand; ^3^Department of Paediatrics, King Chulalongkorn Memorial Hospital, The Thai Red Cross Society, Bangkok 10330, Thailand; ^4^Population, Policy & Practice Research and Teaching Department, University College London Great Ormond Street Institute of Child Health, London, UK

## Abstract

**Introduction:**

Obesity may be associated with poor iron status. The objective of this study was to investigate the association between different indices of iron status and anthropometric measurements in Thai children.

**Materials and Methods:**

Anthropometry (weight, height, waist circumference (WC), and body composition assessed by bioelectrical impedance analysis) and iron indices were measured in 336 Thai children aged 6–12 years. Iron deficiency (ID) was defined using two or more of the following: (1) %transferrin saturation (%Tsat) < 16%; (2) serum ferritin (SF) < 15 *μ*g/mL; and (3) soluble transferrin receptor (sTfR) > 5 mg/L. Iron deficiency anaemia (IDA) was defined as haemoglobin < WHO age cutoff combined with ID. Overweight and obesity were defined as body mass index (BMI) standard deviation score (SDS) ≥ +1 SDS or +2 SDS, respectively (WHO growth reference).

**Results:**

BMI SDS was significantly positively correlated with sTfR and SF (sTfR, *r*: 0.209, *p* < 0.001; SF, *r*: 0.214, *p* < 0.001) and negatively correlated with %Tsat (*r*: −0.132, *p* = 0.013). Correlations between WC SDS and %fat mass and each iron marker were similar. The percentage with low SF was significantly lower than that using other individual markers. ID prevalence was not significantly different between normal-weight and overweight/obesity groups although a significantly higher proportion of overweight/obese children had sTfR >5 mg/L. Puberty and menarche were significant predictors of ID (puberty adjusted OR: 2.20, 95% CI: 0.43, 11.25; menarche adjusted OR: 6.11, 95% CI: 1.21, 30.94).

**Conclusion:**

Greater adiposity was associated with poorer iron status. However, SF may not be a good indicator of iron status in Thai children, particularly in those who are overweight/obese, whereas sTfR merits further investigation.

## 1. Introduction

Thailand is experiencing a double burden from malnutrition characterised by the coexistence of undernutrition and overnutrition in the same individuals, families, or populations [1]. Micronutrient deficiencies remain a major public health challenge, while the prevalence of obesity has increased over the past several decades [2].

Previous studies have shown that obesity is associated with a higher risk of metabolic complications and nutritional deficiencies, especially iron deficiency (ID) [3, 4]. However, the aetiology of the association between obesity and ID remains unclear. Poor iron intake and an increased iron requirement are known to be associated with ID [5, 6]. However, dietary factors alone cannot explain the ID observed in obese individuals [7]. It is now assumed that low-grade chronic inflammation associated with obesity may play an important role in explaining low serum iron levels [4]. The inflammatory state may affect iron absorption and alter iron recycling [8–10].

Since ID in children is associated with adverse outcomes such as poor growth and cognitive dysfunction, prevention and early detection of ID are crucial [11]. However, the optimal method to assess iron status in overweight/obese children might differ from that in normal-weight children due to differences in iron metabolism [12–14]. For example, serum ferritin (SF) which is usually used to determine iron status in community-based cross-sectional studies might be high due to the chronic inflammatory state associated with obesity as mentioned above [4]. Therefore, to identify ID in overweight/obese children, more data are needed regarding the limitations of each iron marker for assessing iron status and the effect of adiposity on each iron index.

Currently, there are few studies on the association between adiposity and different iron indices in school-aged children, and most were performed in Caucasian populations which may differ from the Asian population [15]. The study conducted by Chang et al. showed that Taiwanese overweight and obese children had a lower risk of ID than normal-weight children which differed from the findings of studies in White or Hispanic children. Additionally, lower serum IL-10 and hepcidin were found in overweight/obese Taiwanese children. The authors commented that the decrease in IL-10 and increase in erythropoiesis might be due to haemolysis from *β*-thalassemia, leading to the downregulation of hepcidin. However, the main weakness of this study is the failure to measure Hb and sTfR, which acts as an erythropoietic activity-associated factor, to help interpret the correlation between hepcidin and erythropoiesis. Nonetheless, these findings suggest that the association between adiposity and iron status might differ between Asian and Caucasian or Hispanic populations [15]. Additionally, most studies used body mass index (BMI) as a proxy for adiposity which might be confounded by lean mass.

Therefore, the aim of this study was to investigate associations between each iron index and adiposity measured by different methods such as BMI, waist circumference (WC), and bioelectrical impedance analysis (BIA) in children in Thailand, an area with high prevalence of thalassemia and haemoglobinopathies. This information could contribute to a better understanding of the effect of adiposity on the change in each iron marker and raise clinical awareness in healthcare professionals about the need to provide appropriate dietary recommendations.

## 2. Materials and Methods

A cross-sectional study was performed in Thai children aged 6–12.9 years. The sample size was calculated based on Muzzio et al.'s study which reported that the correlation coefficient between BMI SDS and SF was 0.180 with *p* = 0.025. Therefore, at least 320 children would be required to detect this correlation if the threshold probability for type I error (ꭤ) was 0.05, and the probability for type II error (*β*) was 0.1 [16]. Children were recruited from a primary school in Bangkok and health fair events coordinated by King Chulalongkorn Memorial Hospital (KCMH), Bangkok, Thailand, between 1^st^ October 2016 and 31^st^ May 2018. Those who were diagnosed with any chronic disease or who currently received any iron supplementation were excluded from all analyses. The study was ethically approved by the Human Ethics Committees and Institutional Review Board of Chulalongkorn University, Thailand (IRB no: 512/59).

Written informed consent was obtained from the parents or legal guardian, and written assent was obtained from the child. Demographic data were collected from a self-administered questionnaire which was completed by the child's caregivers, including information about the primary caregivers, family income, underlying diseases, menarche, and the amount of any iron supplements given. Pubertal stage was assessed by paediatricians and classified by using the Tanner stage. Tanner stage equal to or more than 2 was classified as representing puberty, while Tanner stage 1 was categorized as prepuberty.

Dietary intake was recorded by dieticians using 24-hour dietary recall. The food-exchange model developed by the Thai Dietetic Association was used during the interview in order to estimate the portion size for each food group. The data from food records were computed by INMUCAL version 2.0 developed by the Institute of Nutrition, Mahidol University, Thailand, to analyze nutrient values. Nutrient intakes calculated from INMUCAL were compared with Thai dietary recommended intake (DRI) 2003 to determine nutrient adequacy [17].

Weight, height, and waist circumference (WC) were measured by paediatricians. All measurements were performed in duplicate, and the average was used in the analyses. Weight was measured to the nearest 100 grams using an electronic weighing scale (Tanita body composition analyzer, model BC-418, Tokyo, Japan) and height to the nearest 1 millimeter using a stadiometer (Seca, model 220, Hamburg, Germany). BMI was computed as weight (kilograms) divided by height squared (meters). Weight and height were converted into weight-for-age SDS (WFA SDS) and height-for-age SDS (HFA SDS) based on Thai growth standard (1997) data using INMU-NutriStat developed by the Institute of Nutrition, Mahidol University, Thailand. Due to the lack of Thai BMI reference data, BMI SDS was computed using age and sex-specific World Health Organization child growth reference data for 5–19 years using the AnthroPlus V1.0.4. program. BMI SDS ≥+1 SD was categorized as overweight, while BMI SDS ≥+2 SD was classified as obesity.

WC was measured to the nearest 1 millimeter using a nonstretch tape (AnthroFlex, model NA305, Minnesota, USA) at the midpoint between the lower costal margin and the top of the iliac crest. Due to the absence of Thai National WC data, WC SDS was calculated based on the data from the study of Rerksuppaphol S. and Rerksuppaphol L. [18]. WC SDS ≥1.25 SD (90^th^ percentile) was classified as central obesity [19].

To evaluate body composition, whole-body impedance was assessed using bioelectrical impedance analysis (BIA) (Tanita body composition analyzer, model BC-418, Tokyo, Japan) with an operating frequency of 50 kHz at 550 mA. In order to avoid introducing additional error by using equations to estimate fat-free mass (FFM) which might be confounded by body size and age, the impedance was recorded directly and converted into the impedance index by dividing impedance by height squared (in meter). Lean mass SDS was computed from the impedance index based on British reference data because no Thai reference data were available for BIA [20]. However, %FM reported by BIA was also recorded.

Blood samples were obtained for haematological and biological tests between 8.00 am and 10.00 am after a 12-hour overnight fast in order to avoid confounding from meals and diurnal variation and analyzed at a laboratory, KCMH, which was approved as a standard certified laboratory by the Randox International Quality Assessment Scheme, Randox Laboratories Ltd., United Kingdom. Complete blood count was performed by automated haematology analyzers (Sysmex XN-10, Kobe, Japan), while serum iron (SI), SF, transferrin, total iron-binding capacity (TIBC), and soluble transferrin receptor (sTfR) were evaluated using a clinical chemistry analyzer (Cobas c 501, Roche, Penzberg, Germany). All iron indices were measured by calorimetric assay except ferritin, which was determined by electrochemiluminescence immunoassay. %transferrin saturation (%Tsat) was computed by (SI/TIBC) × 100.

To define anaemia, the WHO cutoff for the study's sample age range was applied [21]. Anaemia was diagnosed when haemoglobin (Hb) concentration was below 11.5 g/L and 12 g/L in children aged 5–11 and 12–14 years, respectively. SF and %Tsat cutoff were based on sex-specific thresholds proposed by the WHO. However, no threshold for sTfR has been suggested by the WHO. Therefore, the sTfR cutoff value used in this study was based on the research of Cepeda-Lopez et al. which used the same method for analysing sTfR [14]. However, many confounding factors might affect the accuracy of each of the iron indices. For example, SF might increase during occult infection or inflammation. Also, %Tsat has a 5–30% biological diurnal and day-to-day variation as well as fluctuation after meals [21]. Moreover, sTfR may be altered by increased red cell production or turnover, as a result of thalassemia and hemoglobinopathy, which have a high prevalence in Thailand [21]. Since there is no universal routine screening for thalassemia or hemoglobinopathy in Thailand, children with a mild form of thalassemia or hemoglobinopathy who are typically asymptomatic may be undiagnosed and therefore recruited for this study. For these reasons, ID in this study was defined using two or more of the following: (1) %Tsat less than 16%, (2) SF cutoff less than 15 *μ*g/mL, and (3) sTfR more than 5 mg/L. IDA was defined as an ID with anaemia.

Data were analyzed using IBM SPSS Statistics version 22 (IBM Corp., Armonk, NY, USA). Histograms and Kolmogorov–Smirnov test were performed to evaluate statistical normality of continuous variables. Descriptive data were presented using mean (standard deviation, SD) or median (interquartile range, IQR1-3) for continuous variables as appropriate. Categorical variables were expressed as frequencies and percentages. The chi-squared test was applied to compare categorical variables between groups. Additionally, the Mann–Whitney test or independent *t*-test was employed to evaluate differences in continuous data between the groups as appropriate. Since data for iron indices were skewed, the Spearman test was used to estimate correlations between indices and anthropometric SDS. Other covariable factors including age, sex, family income, pubertal stage, menarche, and iron intake which may be predictors of ID and IDA were examined using univariate and multivariate binary logistic regression. *p* < 0.05 was considered to be statistically significant.

## 3. Results

### 3.1. Demographic Data

A total of 355 participants were enrolled. However, 19 participants were excluded because insufficient blood was obtained to assess iron status. Therefore, data from 336 participants were analyzed. The mean age of all participants was 9.9 ± 1.7 years. 21% and 53% of boys and girls, respectively, were in puberty with considerably more girls than boys at Tanner stages 2–5 (*p* < 0.001). Over 60 percent were from lower-middle-class socioeconomic status with family income ranging from 490 to 1640 US dollars per month. No significant differences were found between boys and girls with regard to the main caregiver and socioeconomic status. Additionally, according to the WHO growth reference, the prevalence of overweight and obesity in the study population was 34%, and the prevalence of central obesity was 16% but higher in boys than girls (22.9% vs. 8.7%, *p* = 0.001) (Table 1).

Regarding dietary intake, the mean total energy and zinc intakes of the children were around 89.1% and 93.4% of the Thai DRI for age and sex, respectively, whereas the protein intake was 1.4 times higher than the recommendation. The mean iron intake was only 59% of the DRI, but there was no difference between the normal-weight and overweight/obesity groups (6.34 ± 3.44 vs. 6.82 ± 3.42 mg/day, *p* = 0.369). Additionally, only 11.9% and 37.1% of the participants had adequate iron and zinc consumption, respectively.

### 3.2. Prevalence of ID and IDA

The prevalence of ID and IDA in the study population was 5.4% and 0.6%, respectively, while the prevalence of all causes of anaemia was 7.3%. There was no significant difference in the prevalence of ID and IDA between the normal-weight and overweight/obese group (ID: 5% vs. 6.1%, *p* = 0.391; IDA: 0.5% vs. 0.9%, *p* = 0.216).

Results for iron status according to the prespecified cutoff values for Hb and each iron index are shown in Table 2. There were no significant differences in the prevalence of anaemia or the percentage with SF or %Tsat lower than the cutoff value between the normal-weight and overweight/obesity group. However, the number of participants with sTfR more than 5 mg/L in the overweight/obesity group was nearly twice as high as in the normal-weight group (31.3% vs. 14.9%, *p* = 0.002).

Furthermore, there was a considerable discrepancy in iron status assessed by ferritin compared with other iron indices. The proportions with ferritin less than 15 *µ*g/L in the normal-weight and overweight/obese groups were only 2.7% and 1.7%, respectively, while other iron markers suggested that the number of study participants with poor iron status was 13.9–31.3%. The agreement between SF and %Tsat or sTfR was low; Cohen's kappa coefficients between SF and %Tsat or sTfR were 0.109 (*p* = 0.003) and 0.037 (*p* = 0.229), respectively. Moreover, there was no difference in the level of agreement between SF and the other iron indices in normal-weight and overweight/obese groups.

### 3.3. Associations between Anthropometry and Each Iron Index

**Figure 1:**
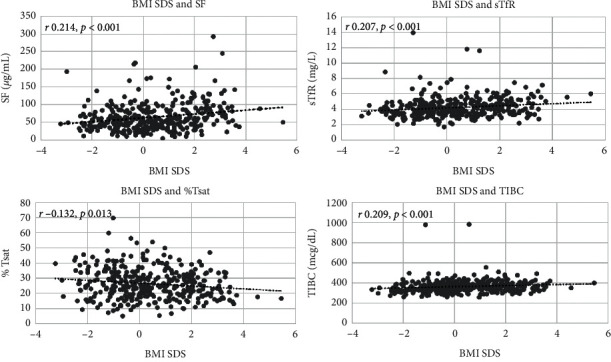
Associations between BMI SDS and SF, %Tsat, and sTfR in 6–12-year-old Thai children (*n* = 336).

Table 3 and Figure 1 show the correlations between each anthropometric measurement and iron index. All anthropometric SDS had a positive correlation with TIBC, SF, transferrin, and sTfR. By contrast, there was a significant negative correlation between %Tsat and the anthropometric SDS.

Higher BMI SDS may significantly correlate with poor iron status, as demonstrated by the positive correlation of BMI SDS with TIBC, transferrin, and sTfR and negative correlation with %Tsat. However, there was a positive correlation between SF and BMI SDS, which might result from a low-grade inflammatory state associated with increased body fat mass. Correlations between %FM or lean mass SDS and each iron index were similar to those for BMI SDS.

### 3.4. Predictors of Iron Deficiency and Iron Deficiency Anaemia

The univariate and multivariate analysis included other covariables including age, sex, family income, pubertal stage, menarche, overweight/obesity, and iron intake in order to examine any prognostic factors associated with ID and IDA (Table 4). Children who were in puberty and girls who had reached menarche had a higher odds ratio (OR) for ID (puberty: crude OR: 4.62, 95% CI: 1.25, 11.23, *p* = 0.02; menarche: crude OR: 8.04, 95% CI: 2.10, 30.79, *p* = 0.002). In multivariate logistic regression analysis, including the factors significantly associated with ID in univariate analyses, menarche was the only statistically significant predictive factor for ID (menarche adjusted OR: 6.11, 95% CI: 1.21, 30.94, *p* = 0.03). Other covariables which have been previously reported to be associated with ID and IDA—age, gender, family income, pubertal stage, overweight/obesity, and inadequate iron intake—were not significantly associated with ID or IDA in our study.

## 4. Discussion

This is the first study performed in Thailand exploring the association between iron markers and anthropometric measures used for determining adiposity in school-age children. The results showed significant associations between body adiposity and each iron marker. Additionally, we also found that SF may not be a good indicator for screening for ID in Thai children.

In this study, when analyzed as continuous variables, measures of adiposity were associated with iron indices. Both WC and %FM had a significant positive correlation with SF, TIBC, transferrin, and sTfR, while there was a negative correlation between WC SDS, %FM, and %Tsat. Similarly, the correlation between BMI SDS and each iron marker was in the same direction as WC SDS and %FM. These correlations were weak and must be interpreted with caution. Nonetheless, these results are consistent with previous research [3, 5, 13–16, 22–27]. For example, a cross-sectional study conducted by Cepeda-Lopez et al. [14] showed a positive correlation between BMI SDS and sTfR (*r*: 0.240, *p*: 0.009), and a cross-sectional study from Taiwan showed a positive correlation between BMI and SF (*r* = 0.21, *p* < 0.001) but a negative correlation between BMI and SI (*r* = −0.057, *p* < 0.001) [28]. These findings suggest that overweight and obese children's iron metabolism differs from that of normal-weight children. Low-grade chronic inflammation associated with obesity may stimulate hepcidin synthesis which is a key hormone regulating iron homeostasis [13, 29, 30]. Consequently, there is impaired duodenal iron absorption and increased sequestration of iron in obese individuals [9, 10]. Transferrin and transferrin receptor increase to bind and deliver iron to cells in order to maintain iron homeostasis. Therefore, overweight/obese children may require higher iron intakes. The increase in SF found in overweight/obese children reflects the fact that SF is also an acute-phase protein whose levels are elevated in response to obesity-induced inflammation [31, 32]. Thus, higher SF in overweight/obese children does not indicate higher iron stores.

In contrast, when adiposity was categorized as normal weight and overweight/obese and iron status was classified as ID and IDA, we found no significant difference in the prevalence of ID or IDA between the two weight groups. This finding is contrary to previous studies which have suggested that overweight/obesity may increase the risk of ID. A recent meta-analysis reported that overweight and obese children had a significantly increased risk of ID (OR: 1.31, 95% CI: 1.01–1.68) [6]. A possible explanation for our result may be a lack of statistical power when the data were analyzed as categorical variables because the sample size of the current study was relatively smaller than the previous studies which reported a difference in the prevalence of ID and IDA between normal-weight and overweight/obese children [3, 15]. Moreover, the previous meta-analysis also reported that the association between ID and overweight/obesity may depend on the method used to diagnose ID. No correlation between ID and obesity was found in eight studies which used a ferritin-based diagnosis (OR: 1.04, 95% CI: 0.69–1.56), whereas using nonferritin-based diagnosis, overweight and obese children were at 49% more risk of ID (OR: 1.49, 95% CI: 1.19, 1.85) [6]. These findings are consistent with previous studies in adults which showed that ferritin-based diagnosis may have limitations for the diagnosis of ID in obese patients [33, 34]. Similarly, in our study, which defined ID by using multiple markers including SF, we did not find any correlation between ID and overweight/obesity.

Our results suggest that a SF cutoff of less than 15 *μ*g/mL as recommended by the WHO showed poor sensitivity for screening for ID in Thai children compared to other markers, as the prevalence of ID using this SF cutoff was significantly lower than that using other single markers. Additionally, there was poor agreement between SF and %Tsat or sTfR for assessing iron status. The most likely explanation for the poor sensitivity of SF in our study may be the high prevalence of occult infection/inflammation in Thai school-age children. A survey in 1,909 Thai students showed that nearly 40% of children were infested with intestinal parasites [35]. Also, the WHO suggested that, in population surveys, if the percentage of SF below the cutoff is less than 20% while the percentage of sTfR above the cutoff is higher than 10%, as in our study, it can be assumed that iron deficiency and inflammation are prevalent [36]. Further studies are required to define the appropriate SF cutoffs in Thai children.

sTfR is a sensitive indicator of iron stores that is unaffected by infection or inflammation [36], while other iron indices including SI, TIBC, and SF are profoundly affected by inflammation [32]. However, sTfR is affected by the rate of erythropoiesis [36]. Our study found that sTfR may have a higher sensitivity for screening for ID in Thai children, especially in overweight/obese children. The prevalence of ID determined by sTfR in overweight/obese children was significantly higher than the normal-weight group and also double that assessed by %Tsat. However, since thalassemia and hemoglobinopathy cannot be excluded in our cohort, the results could also be affected by haemolysis.

The current study also found that puberty and menarche were associated with increased OR for ID. This result may be explained by the fact that the accelerated growth during the pubertal period leads to an increase in blood volume, red cell mass, and lean body mass, resulting in an increase in iron demand for Hb in blood and myoglobin in muscles [37]. Moreover, in girls, blood loss from menstruation can result in depletion of the iron stores [38].

The strengths of this study are that it is the first to explore the associations between body adiposity and iron markers in Thai children. It also applied multiple iron indices to determine iron status which could increase the validity of the assessment. Moreover, the study used various indicators to measure body adiposity. However, there were several limitations. Firstly, there was a lack of data on skinfold thickness or whole-body DEXA which would be better simple measurement methods to assess FM [39]. Secondly, this study did not evaluate any inflammatory markers or thalassemia profile which may affect the concentration of different iron markers. Finally, the data in this study were collected from only one province in Thailand which might limit the generalizability of the findings to the general population. Further research should be performed to investigate the appropriated SF cutoff level for Thai children. Ideally, future studies should include haemoglobin electrophoresis, measurement of inflammatory markers, and details of body adiposity assessed by other methods such as skinfold thickness or whole-body DEXA.

## 5. Conclusion

This study found that an increase in adiposity correlated with poorer iron status which was demonstrated by lower SI and %Tsat and higher TIBC and sTfR. Overweight/obese children may require higher iron intake to maintain their iron homeostasis. Additionally, we found that SF may not be a good indicator for determining iron status in overweight/obese Thai children. This information can contribute to the development of appropriate guidance for screening for ID in overweight and obese children.

## Figures and Tables

**Table 1:** Characteristics of the study participants.

	Boys (%) (*n* = 182)	Girls (%) (*n* = 154)	Total (%) (*n* = 336)
Age (years) ^†^			
(i) 6–<9	29.1	31.2	30.1
(ii) 9–12	70.9	68.8	69.9

Tanner stage			
(i) Stage 1	78.6	46.8^∗^	64.0
(ii) Stage 2	11.5	25.3	17.5
(iii) Stage 3	6.6	17.5^∗^	11.6
(iv) Stage 4	2.7	9.8^∗^	6.0
(v) Stage 5	0.5	0.6	0.6

Menarche			
(i) Yes	—	15.9	—
(ii) No	—	84.1	—

Main caregiver			
(i) Mother	69.6	76.9	73.3
(ii) Father	15.2	10.3	12.7
(iii) Others	15.2	12.8	14.0

Family income (USD/month)			
(i) <490	20.7	27.4	24.1
(ii) 490–985	37.8	28.2	32.9
(iii) 985–1640	27.0	29.1	28.1
(iv) 1640–3290	10.8	12.8	11.8
(v) >3290	3.6	2.6	3.1

Nutritional status ^‡^			
(i) Moderate and severe wasting	1.1	0	0.6
(ii) Mild wasting	3.8	7.8^∗^	5.7
(iii) Normal	52.2	68.2^∗^	59.5
(iv) Overweight	17.6	14.3	16.1
(v) Obesity	25.3	9.7^∗^	18.2

Central adiposity ^§^			
(i) Yes	22.9	8.7^∗^	16.1
(ii) No	77.1	91.3^∗^	83.9

^*∗*^*p* < 0.05, significantly different from boys. ^†^Children aged 6–8.99 years were placed in the group of 6–<9 years old, while children aged 9–12.99 years were placed in the group of 9–12 years old. ^‡^Nutritional status was classified by BMI SDS according to the WHO growth reference. ^§^WC SDS ≥1.25 SD (90^th^ percentile) calculated based on the data from the study of Rerksuppaphol S. and Rerksuppaphol L. was classified as central obesity [18].

**Table 2:** Prevalence of iron deficiency and anaemia using different indices.

	Normal weight (%) (*n* = 200)	Overweight/obesity (%) (*n* = 115)	Total (%) (*n* = 315)
Anaemia	9.2	3.5	7.3
SF less than 15 *μ*g/mL	2.7	1.7	2.4
%Tsat less than 16%	14.0	13.9	14.0
sTfR more than 5 mg/L	14.9	31.3^∗^	20.5

^*∗*^*p* < 0.05, significantly different from the normal-weight group.

**Table 3:** Correlation coefficients between each iron index and anthropometry.

	Hb	SF	SI	TIBC	Transferrin	%Tsat	sTfR
WFA SDS	0.083	0.163^∗∗∗^	−0.098^∗^	0.254^∗∗^	0.254^∗∗^	−0.160^∗∗^	0.249^∗∗^
HFA SDS	0.130^∗^	0.076	−0.102^∗^	0.221^∗∗^	0.217^∗∗^	−0.154^∗∗^	0.216^∗∗^
BMI SDS	0.031	0.214^∗∗^	−0.062	0.207^∗∗^	0.211^∗∗^	−0.132^∗^	0.209^∗∗^
WC SDS	0.030	0.198^∗∗^	−0.110^∗^	0.174^∗∗^	0.166^∗∗^	−0.153^∗∗^	0.252^∗∗^
%FM by BIA	0.029	0.193^∗∗^	−0.033	0.213^∗∗^	0.218^∗∗^	−0.095	0.160^∗∗^
Lean mass SDS	0.118^∗^	0.081	−0.103	0.277^∗∗^	0.281^∗∗^	−0.179^∗∗^	0.266^∗∗^

^∗^Correlation is significant at the 0.05 level (2-tailed). ^∗∗^Correlation is significant at the 0.01 level (2-tailed).

**Table 4:** The predictors of iron deficiency and iron deficiency anaemia.

	Iron deficiency				Iron deficiency anaemia			
	Crude OR (95% CI)	*p*	Adjusted OR (95% CI)	*p*	Crude OR (95% CI)	*p*	Adjusted OR (95% CI)	*p*
Age (yr)	1.36 (1, 1.80)	0.06			1.10 (0.47, 2.57)	0.817		
Gender								
(i) Male	1				1			
(ii) Female	1.19 (0.54, 3.13)	0.55			1.18 (0.07, 19.07)	0.91		

Family income (USD)								
(i) ≤490	1				1			
(ii) 490–985	1.25 (0.35, 4.54)	0.69			1 (0, 1)	0.99		
(iii) 985–1640	0.41 (0.09, 2.43)	0.32			1 (0, 1)	1.00		
(iv) 1640–3290	0.43 (0.05, 2.33)	0.45			1 (0, 1)	1.00		
(v) 3290	0 (0, 1)	0.99			1 (0, 1)	1.00		

Pubertal stage								
(i) Prepubertal stage	1		1		1			
(ii) Pubertal stage	4.621 (1.25, 11.23)	0.02^∗^	2.20 (0.43, 11.25)	0.056	1.16 (0.07, 18.77)	0.92		
Menarche								
(i) No	1		1		1			
(ii) Yes	8.04 (2.10, 30.79)	<0.01^∗^	6.11 (1.21, 30.94)	0.03^∗^	11.94 (0.71, 199.46)	0.08		

Nutritional status								
(i) Normal weight	1				1			
(ii) Overweight/obesity	1.24 (0.47, 3.22)	0.67			1.93 (0.12, 31/14)	0.64		

Iron intake								
(i) Adequate iron intake	1				1			
(ii) Inadequate iron intake	0.66 (0.08, 5.31)	0.69			0 (0, 1)	0.99		

## Data Availability

The data that support the findings of this study are available from the initial author upon request.
